# Ambient Coarse Particulate Matter and Human Health: A Systematic Review and Meta-Analysis

**DOI:** 10.1007/s40572-014-0022-z

**Published:** 2014-08-08

**Authors:** Sara D. Adar, Paola A. Filigrana, Nicholas Clements, Jennifer L. Peel

**Affiliations:** 1 Department of Epidemiology, University of Michigan, School of Public Health, 1420 Washington Heights – SPHII-5539, Ann Arbor, MI 48109-2029 USA; 2 Department of Mechanical Engineering, University of Colorado, 135 30th St., Boulder, CO 80305 USA; 3 Department of Environmental and Radiological Health Sciences, Colorado State University, Campus Delivery 1681, Fort Collins, CO 80523-1681 USA

**Keywords:** Air pollution, Coarse particulate matter, Health, Cardiovascular, Respiratory, Mortality, Hospitalizations, Time-series, Case-crossover

## Abstract

Airborne particles have been linked to increased mortality and morbidity. As most research has focused on fine particles (PM_2.5_), the health implications of coarse particles (PM_10-2.5_) are not well understood. We conducted a systematic review and meta-analysis of associations for short- and long-term PM_10-2.5_ concentrations with mortality and hospital admissions. Using 23 mortality and 10 hospital admissions studies, we documented suggestive evidence of increased morbidity and mortality in relation to higher short-term PM_10-2.5_ concentrations, with stronger relationships for respiratory than cardiovascular endpoints. Reported associations were highly heterogeneous, however, especially by geographic region and average PM_10-2.5_ concentrations. Adjustment for PM_2.5_ and publication bias resulted in weaker and less precise effect estimates, although positive associations remained for short-term PM_10-2.5_ concentrations. Inconsistent relationships between effect estimates for PM_10-2.5_ and correlations between PM_10-2.5_ and PM_2.5_ concentrations, however, indicate that PM_10-2.5_ associations cannot be solely explained by co-exposure to PM_2.5_. While suggestive evidence was found of increased mortality with long-term PM_10-2.5_ concentrations, these associations were not robust to control for PM_2.5_. Additional research is required to better understand sources of heterogeneity of associations between PM_10-2.5_ and adverse health outcomes.

## Introduction

Airborne particulate matter has been consistently linked to adverse health, including mortality and morbidity from respiratory and cardiovascular diseases [1]. As particles less than 10 μm in aerodynamic diameter (PM_10_) can reach the tracheobronchial and alveolar regions of the respiratory tract [2], these particles have been of prime interest for epidemiology studies. PM_10_ is comprised of two distinct types of particles with different morphologies and sources. Fine particles, < 2.5 μm (PM_2.5_), are typically generated by combustion or photochemical reactions in the atmosphere and are thus generally comprised of organic carbon, elemental carbon, sulfate, nitrate, and metals. In contrast, coarse particles (typically classified as 2.5–10 μm, PM_10-2.5_) are commonly formed by mechanical grinding and resuspension of solid material. This results in a primary composition of crustal elements, metals from suspended road dust, and organic debris [3–5]. These variations in composition, along with differential deposition in the body [2], suggest that PM_2.5_ and PM_10-2.5_ may differ in their impacts on human health.

To date, the vast majority of research has focused on PM_2.5_ or PM_10_; far less is known about the health implications of PM_10-2.5_. This represents a critical gap in our understanding with direct policy implications. For example, the United States Environmental Protection Agency (EPA) has stated that PM_2.5_ and PM_10-2.5_ should be considered separately under the National Ambient Air Quality Standards (NAAQS), but a unique PM_10-2.5_ standard has not yet been adopted. Rather, PM_10-2.5_ is regulated through the PM_10_ standard. This approach has been attributed in part due to the sparse epidemiological data available examining associations between exposures to PM_10-2.5_ and health effects [5].

Over the past decade, an increasing number of epidemiological investigations have explored PM_10-2.5_-related health effects. As reviewed by Brunekreef and Forsberg in 2005 [6], early evidence suggested the presence of associations for morbidity and mortality with short- but not long-term exposures to PM_10-2.5_. Associations were noted to differ by location, with stronger associations in more arid locations. Associations with respiratory hospitalizations were also notably as strong or stronger for PM_10-2.5_ than for PM_2.5_. Since PM_10-2.5_ associations were found to be sensitive to control for PM_2.5_ in the few studies reporting adjusted results, the authors encouraged future research to report multi-pollutant models.

This manuscript extends the work of Brunekreef and Forsberg [6] by incorporating newly published studies on PM_10-2.5_ with mortality and hospitalizations and conducting meta-analyses to generate summary estimates for relationships with PM_10-2.5_. To better understand factors that may modify associations between PM_10-2.5_ and health, we also explored heterogeneity by study location, lag period, ambient concentrations of pollution, the relative abundance of PM_10-2.5_ to PM_2.5_, and sampling methodology for PM_10-2.5_. We further investigated the impact of PM_2.5_ concentrations on associations with PM_10-2.5_ by summarizing results from multi-pollutant models and exploring how the magnitude of association between PM_10-2.5_ and health vary according to correlations between PM_2.5_ and PM_10-2.5_ concentrations.

## Methods

A systematic review was conducted to identify all published studies of short- and long-term exposures to PM_10-2.5_ (or PM_15-2.5_) that reported associations with mortality or hospital admissions. We also compiled data for emergency department visits but restricted these papers to sensitivity analyses to focus our estimates on the most severe health endpoints. Literature searches using the Web of Knowledge and Medline were conducted with the key words “coarse particulate matter” or “PM_10-2.5_” and “health” through the end of December 2013. This approach was supplemented by a review of the reference lists of any identified publications, as well as earlier reviews by the Environmental Protection Agency [5] and Brunekreef and Forsberg [6].

Effect estimates and confidence intervals were extracted from each published report as well as descriptive information about the population, time period, outcome, and exposures. When data or results were discussed but not quantified, we contacted the authors for additional information. Papers were excluded if they did not report or we could not obtain effect estimates for PM_10-2.5_ with concurrent standard errors, confidence intervals, or t-values. When more than one study was available for the same population, we selected the report with the longest follow-up. Since associations for the case-crossover design are mathematically equivalent to those from time-series studies [7], we have used both designs in our meta-analyses, though we have restricted selection to papers employing a time-stratified referent selection strategy due to known bias with other designs [8]. When both case-crossover and time series approaches were presented, the time-series point estimates were included in our meta-analyses. Time-series analyses using non-parametric smoothing splines (except penalized splines) and generalized additive models in S-Plus were also excluded based on previously identified issues with model convergence and the underestimation of standard errors [9]. Citations were identified and summarized independently by two investigators.

To be included in our quantitative meta-analysis, five or more studies were required for a particular health endpoint. We identified associations a priori with the previous day (Lag 1), current day (Lag 0), and two days prior (Lag 2) as our primary analyses for total mortality, cardiovascular endpoints, and respiratory endpoints, respectively. When these exact lags were unavailable, we selected the next closest time point. All associations were standardized to a difference of 10 μg/m^3^ and summarized across investigations using meta-analysis (STATA v13, Stata Corp, College Station, TX). To account for heterogeneity across studies, we employed the DerSimonian and Liard random effects approach and report the I^2^ statistic as an indicator of the fraction of the variability due to true between-study differences as opposed to chance [10]. Publication bias was also explored using funnel plots, Egger’s test of asymmetry [11], and the “trim and fill” approach to estimate the associations that might have been observed in the absence of publication bias [12].

To explore possible causes for heterogeneity in effect estimates, we conducted analyses stratified by geographic location and lag period. We also examined non-linearity of the dose-response relationship through stratification by PM_10-2.5_ concentrations and meta-regression. Differences in associations by PM_2.5_ concentrations and the ratio of PM_10-2.5_ to PM_10_ were similarly explored to assess if PM_10-2.5_ from regions with more urban/industrial pollution from combustion had greater toxicity than PM_10-2.5_ from other settings. In addition, we summarized all available associations with PM_10-2.5_ adjusted for PM_2.5_ and investigated if PM_10-2.5_ associations were greater in locations with higher correlations between PM_10-2.5_ and PM_2.5_ concentrations. Finally, we explored if sampling methods suspected to have more (i.e., tapered element oscillating microbalance, TEOM) or less measurement error (i.e., dichotomous sampler) for PM_10-2.5_ [13] were found to impact associations.

## Results

### Papers Identified with Short-Term PM_10-2.5_ Exposures

A total of 34 published studies were identified that presented associations between short-term fluctuations in PM_10-2.5_ concentrations and mortality. Of these investigations, we excluded three manuscripts with incomplete reporting of numerical results [14–16]. An additional nine papers were excluded for use of non-parametric smoothing splines in GAM. Of these, seven [17–23] were replaced by later re-analysis of the same data [9], but two were without replication [24, 25]. Similarly, three papers were superseded by longer time series from the same populations [22, 26, 27], and one was excluded, as it was a sensitivity analysis of another report [28]. One final paper was excluded as it only explore stroke mortality [29]. This resulted in 23 studies for inclusion in this meta-analysis—19, 11, and 14 total cases of non-accidental [9, 30–32, 33••, 34, 35, 36••, 37, 38, 39••, 40–45], respiratory, [9, 33••, 34, 36••, 37, 38, 39••, 41, 42, 46, 47], and cardiovascular mortality [9, 33••, 34, 35, 36••, 37, 38, 39••, 41, 42, 46, 47], respectively. No other cause-specific mortality had sufficient counts to be included.

For hospital admissions, we identified 23 studies and one scientific report with published associations for short-term exposures to PM_10-2.5_. Of these investigations, we excluded eight manuscripts for using non-parametric smoothing splines in GAM or case-crossover reference strategies inconsistent with current recommendations [18, 24, 48–53]. Two of these investigations [18, 53] were re-analyzed [9], and therefore included in our analysis. An additional study was excluded for using an ordinary least squares approach for time-series [54], two as sensitivity analyses of primary results presented elsewhere [55, 56] and another four for including health outcomes with insufficient counts for meta-analysis [18, 52, 57, 58]. After these exclusions, there were a total of 10 papers for meta-analysis, resulting in sufficient counts to explore respiratory (n = 9) [42, 47, 59–61, 62••, 63••, 64, 65•] and cardiovascular hospitalizations (n = 6) [42, 47, 61, 62••, 64, 66]. An additional 12 papers [15, 35, 67–76] and one report [77] were identified on emergency department visits, although these included some extensions of earlier papers and some unique health outcomes that were not reported in a sufficient number of studies to support meta-analysis.

Table 1 summarizes the studies included in this meta-analysis. Across all of the investigations of short-term exposures to PM_10-2.5_, a total of 9.3 million non-accidental deaths, 0.75 million respiratory deaths, and 2.4 million cardiovascular deaths were enumerated. Additionally there were 2.8 and 5.4 million hospital admissions for respiratory and cardiovascular causes, respectively. Most of these investigations (80 %) utilized a time-series design and were conducted in either North America or Europe. In the regions studied, concentrations of PM_10-2.5_ and PM_2.5_ ranged from lows of 3.7 and 6.7 μg/m^3^ in the United States to highs of 101 and 94 μg/m^3^ in China, respectively. Correlations between these two pollutants were generally modest and ranged from -0.03 in the United States to 0.73 in France.

**Table 1:** Descriptive information for short-term exposure studies included in the meta-analysis

Study	Location	Time Period	Study Design	Restrictions	Reported or Estimated # of Events (Short-Term) or # of Participants (Long-Term)	Estimated Incidence Rate Ratios (95 % CI) per 10 μg/m^3^ of PM_10–2.5_	Estimated Incidence Rate Ratios (95 % CI) per 10 μg/m^3^ of PM_2.5_	Median or Mean† PM_10–2.5_	Median or Mean† PM_2.5_	Correlation of PM_10–2.5_ and PM_2.5_
Short-Term Associations With Non-Accidental Mortality										
Atkinson et al. 2010	London, United Kingdom	2000–2005	Time-Series		278,545	1.018 (1.007, 1.030)	1.000 (0.996, 1.004)	7.0	15	0.22
Burnett et al. 2004	12 Cities, Canada	1981–1999	Time-Series		1,450,251	1.006 (0.999, 1.014)	1.005 (0.991, 1.020)	11.4†	12.8†	
Chen et al. 2011	3 Cities, China	2004–2008*	Time-Series		308,904	1.003 (1.001, 1.004)	1.003 (1.002, 1.004)	49–101†	55–94†	0.28–0.53
Chock et al. 2000	Allegheny County, United States	1989–1991	Time-Series	<75 years	25,609	1.003 (0.993, 1.013)	1.010 (0.992, 1.028)			
Chock et al. 2000	Allegheny County, United States	1989–1991	Time-Series	>75 years	25,109	1.005 (0.995, 1.015)	1.006 (0.988, 1.025)			
Cifuentes et al. 2000	Santiago metropolitan area, Chile	1988–1996	Time-Series		165,668	1.006 (1.001, 1.012)	1.005 (1.003, 1.008)	44.3	42.6	0.52
Fairley 1999/HEI 2003	Santa Clara, United States	1986–1996	Time-Series		58,440	0.978 (0.922, 1.037)	0.984 (0.962, 1.007)	11†	9†	0.51
Janssen et al. 2013	All Cities, Netherlands	2008–2009	Time-Series		258,159	0.998 (0.987, 1.010)	1.008 (1.003, 1.012)	7.2	13.1	0.29
Klemm et al. 2004	Atlanta, United States	1998–2000	Time-Series	>65 years	10,841	1.006 (0.999, 1.014)	1.003 (1.001, 1.005)	9.3	18.1	
Lippmann et al. 2000/HEI 2003	Detroit, United States	1992–1994	Time-Series		25,970	1.011 (0.991, 1.032)	1.008 (0.993, 1.023)	12	15	0.42
Lopez-Villarrubia et al. 2012	Las Palmas de Gran Canaria, Canary Islands	2001–2004	Time-Series		10,811	1.004 (0.981, 1.028)	0.994 (0.959, 1.029)	14.6	12.7	0.55
Lopez-Villarrubia et al. 2012	Santa Cruz de Tenerife, Canary Islands	2001–2004	Time-Series		6,428	1.004 (0.981, 1.028)	0.994 (0.959, 1.029)	20.3	11.3	0.55
Malig et al. 2009	15 California Counties, United States	1999–2005	Case-Crossover		107,188	1.000 (0.989, 1.012)		10.6–46.5†	11.1–17.3†	-0.03–0.35
Mallone et al. 2011	Rome, Italy	2001–2004	Case-Crossover		80,423	1.027 (1.011, 1.044)	1.010 (0.995, 1.025)	13.6, 18.3††	20.9, 24††	0.27, 0.18††
Meister et al. 2012	Stockholm, Sweden	2000–2008	Time-Series		93,398	1.017 (1.002, 1.032)	1.015 (1.001, 1.028)	7.1†	8.6†	0.27
Perez et al. 2008	Barcelona, Spain	2003–2004	Case-Crossover		24,850	1.027 (1.008, 1.046)	1.040 (1.023, 1.057)	12.9	22.4	0.33
Samoli et al. 2013	8 metropolitan areas, European Mediterranean	2001–2010*	Time-Series		578,191	1.003 (0.999, 1.007)	1.006 (1.003, 1.008)	8.0–15.8	13.6–27.7	0.19–0.68
Schwartz et al. 1996/HEI 2003	6 Cities, United States	1979–1988	Time-Series	>65 years	103,841	1.001 (0.995, 1.007)	1.008 (1.004, 1.013)	9	14.7	0.23–0.69
Tobias et al. 2011	Madrid, Spain	2003–2005	Case-Crossover	Dust Days	12,993	1.005 (0.987, 1.026)	1.008 (0.980, 1.040)	22	24	
Tobias et al. 2011	Madrid, Spain	2003–2005	Case-Crossover	Dust-Free Days	53,997	1.021 (1.007, 1.035)	1.030 (1.015, 1.043)	12	16	
Villeneuve et al. 2003	Vancouver, Canada	1986–1998	Time-Series	>65 years	28,210	0.990 (0.964, 1.016)	1.013 (0.983, 1.044)	7.8†	10.7†	0.46
Zanobetti et al. 2009	47 Cities, United States	1999–2005	Time-Series		5,609,349	1.005 (1.002, 1.007)	1.010 (1.008, 1.012)	3.7–33.1†	6.7–21.7†	
Short-Term Associations With Respiratory Mortality										
Atkinson et al. 2010	London, United Kingdom	2000–2005	Time-Series		42,262	1.001 (0.972, 1.031)	1.009 (0.999, 1.019)	7.0	15	0.22
Chen et al. 2011	3 Cities, China	2004–2008*	Time-Series		33,871	1.002 (0.996, 1.008)	1.002 (0.999, 1.005)	49–101†	55–94†	0.28–0.53
Halonen et al. 2009	Helsinki metropolitan area, Finland	1998–2004	Time-Series		3,701	1.005 (0.958, 1.054)	1.000 (0.952, 1.051)	7.5	9.5	0.25
Janssen et al. 2013	All Cities, Netherlands	2008–2009	Time-Series		27,759	1.038 (1.006, 1.072)	1.016 (1.004, 1.029)	7.2	13.1	0.29
Lippmann et al 2000/HEI 2003	Detroit, United States	1992–1994	Time-Series		12,250	1.025 (0.959, 1.096)	1.012 (0.960, 1.067)	12	15	0.42
Lopez-Villarrubia et al. 2012	Las Palmas de Gran Canaria, Canary Islands	2001–2004	Time-Series		979	1.060 (0.987, 1.137)	1.059 (0.948, 1.184)	14.6	12.7	0.55
Lopez-Villarrubia et al. 2012	Santa Cruz de Tenerife, Canary Islands	2001–2004	Time-Series		584	1.060 (0.987, 1.137)	1.059 (0.948, 1.184)	20.3	11.3	0.55
Mallone et al. 2011	Rome, Italy	2001–2004	Case-Crossover		4,574	1.117 (1.011, 1.233)	1.002 (0.922, 1.089)	13.6, 18.3††	20.9, 24††	0.27, 0.18††
Perez et al. 2012	Barcelona, Spain	2003–2007	Case-Crossover	Dust Days	540	1.035 (0.918, 1.167)	1.020 (0.909, 1.145)	11.5	17.3	0.01
Perez et al. 2012	Barcelona, Spain	2003–2007	Case-Crossover	Dust-Free Days	5,812	1.048 (1.013, 1.085)	1.028 (0.994, 1.062)	12.4	19.2**	0.01**
Samoli et al. 2013	8 metropolitan areas, European Mediterranean	2001–2010*	Time-Series		58,440	1.007 (0.997, 1.018)	1.016 (1.006, 1.027)	8.0–15.8	13.6–27.7**	0.19–0.68**
Villeneuve et al. 2003	Vancouver, Canada	1986–1998	Time-Series	>65 years	3,765	1.001 (0.942, 1.063)	1.002 (0.919, 1.092)	7.8†	10.7†	0.46
Zanobetti et al. 2009	47 Cities, United States	1999–2005	Time-Series		547,660	1.012 (1.004, 1.019)	1.017 (1.010, 1.023)	3.7–33.1†	6.7–21.7†	
Short-Term Associations With Cardiovascular Mortality										
Atkinson et al. 2010	London, United Kingdom	2000–2005	Time-Series		103,734	0.996 (0.977, 1.015)	1.001 (0.994, 1.007)	7.0	15	0.22
Chen et al. 2011	3 Cities, China	2004–2008*	Time-Series		126,988	1.001 (1.000, 1.003)	1.005 (1.004, 1.007)	49–101†	55–94†	0.28–0.53
Halonen et al. 2009	Helsinki metropolitan area, Finland	1998–2004	Time-Series		16,233	1.000 (0.979, 1.021)	1.012 (0.989, 1.035)	7.5	9.5	0.25
Janssen et al. 2013	All Cities, Netherlands	2008–2009	Time-Series		78,675	0.981 (0.961, 1.001)	1.011 (1.002, 1.019)	7.2	13.1	0.29
Lippmann et al. 2000/HEI 2003	Detroit, United States	1992–1994	Time-Series		1,960	1.024 (0.994, 1.055)	1.008 (0.986, 1.030)	12	15	0.42
Lopez-Villarrubia et al. 2012	Las Palmas de Gran Canaria, Canary Islands	2001–2004	Time-Series		2,338	1.023 (0.976, 1.072)	1.026 (0.956, 1.101)	14.6	12.7	0.55
Lopez-Villarrubia et al. 2012	Santa Cruz de Tenerife, Canary Islands	2001–2004	Time-Series		1,315	1.023 (0.976, 1.072)	1.026 (0.956, 1.101)	20.3	11.3	0.55
Malig et al. 2009	15 California Counties, United States	1999–2005	Case-Crossover		45,036	1.003 (0.988, 1.017)		10.6–46.5†	11.1–17.3†	–0.03–0.35
Mallone et al. 2011	Rome, Italy	2001–2004	Case-Crossover		24,773	1.034 (1.007, 1.062)	1.011 (0.987, 1.035)	13.6, 18.3††	20.9, 24††	0.27, 0.18
Mar et al 2000/2003	Maricopa County, United States	1995–1997	Time-Series		4,182	1.024 (1.003, 1.046)	1.040 (0.984, 1.100)	33.5†	13.0†	0.5–0.59
Ostro et al. 2000/2003	Coachella Valley, United States	1989–1998	Time-Series		8,073	1.011 (1.002, 1.020)	0.944 (0.882, 1.010)	30.5†	16.8†	0.28
Perez et al. 2012	Barcelona, Spain	2003–2007	Case-Crossover	Dust Days	1,650	1.104 (1.031, 1.181)	1.041 (0.968, 1.122)	11.5	17.3**	0.01**
Perez et al. 2012	Barcelona, Spain	2003–2007	Case-Crossover	Dust-Free Days	16,513	1.041 (1.018, 1.066)	1.030 (1.006, 1.054)	12.4	19.2**	0.01**
Samoli et al. 2013	8 metropolitan areas, European Mediterranean	2001–2010*	Time-Series		213,306	1.003 (0.996, 1.009)	1.006 (1.001, 1.011)	8.0–15.8	13.6–27.7	0.19–0.68
Villeneuve et al. 2003	Vancouver, Canada	1986–1998	Time-Series	>65 years	11,518	1.053 (1.010, 1.098)	0.990 (0.942, 1.041)	7.8†	10.7†	0.46
Zanobetti et al. 2009	47 Cities, United States	1999–2005	Time-Series		1,787,078	1.003 (1.000, 1.006)	1.009 (1.005, 1.012)	3.7–33.1†	6.7–21.7†	
Short-Term Associations With Respiratory Hospitalizations										
Alessandrini et al. 2013	Rome, Italy	2001–2004	Time-Series	<14 years	11,157	0.986 (0.935, 1.038)	0.999 (0.958, 1.041)	14.6 to 20.7††	23.4 to 25.6††	0.25
Alessandrini et al. 2013	Rome, Italy	2001–2004	Time-Series	>35 years	20,463	1.041 (1.004, 1.079)	0.997 (0.969, 1.025)	14.6 to 20.7††	23.4 to 25.6††	0.25
Atkinson et al. 2010	London, United Kingdom	2000–2005	Time-Series	<14 years	67,235	0.998 (0.973, 1.024)	1.017 (1.009, 1.025)	7.0	15	0.22
Atkinson et al. 2010	London, United Kingdom	2000–2005	Time-Series	>65 years	121,023	1.007 (0.988, 1.026)	1.009 (1.003, 1.016)	7.0	15	0.22
Chen et al. 2005	Vancouver, Canada	1995–1999	Time-Series	>65 years	12,880	1.123 (1.048, 1.201)	1.051 (0.975, 1.157)	5.6†	7.7†	0.38
Halonen et al. 2009	Helsinki metropolitan area, Finland	1998–2004	Time-Series		26,095	0.999 (0.981, 1.017)	1.023 (1.004, 1.042)	7.5	9.5	0.25
Host et al. 2008	6 French cities, France	2000–2003*	Time-Series	<14 years	56,387	1.062 (1.004, 1.123)	1.004 (0.988, 1.020)	7.0–11.0†	13.8–18.8†	0.28–0.73
Host et al. 2008	6 French cities, France	2000–2003*	Time-Series	15–64 years	57,589	1.026 (0.995, 1.058)	1.008 (0.993, 1.023)	7.0–11.0†	13.8–18.8†	0.28–0.73
Host et al. 2008	6 French cities, France	2000–2003*	Time-Series	>65 years	56,267	1.019 (0.981, 1.059)	1.005 (0.980, 1.030)	7.0–11.0†	13.8–18.8†	0.28–0.73
Peng et al. 2008	108 Counties, United States	1999–2005	Time-Series	>65 years	1.4 M	0.999 (0.994, 1.005)	1.004 (1.001, 1.008)	13.5	9.8	0.12
Qiu et al. 2012	Hong Kong, Special Administrative Region of China	2000–2005	Time-Series		518,864	1.009 (1.004, 1.014)		14.5	34.8	0.68
Stafoggia et al. 2013	6 metropolitan areas, European Mediterranean	2001–2010	Time-Series	>15 years	459,261	1.012 (0.989, 1.036)	1.011 (1.000, 1.021)	9.3–17.5†	17.2–34.4†	0– > 0.5
Yang et al. 2004	Vancouver, Canada	1995–1999	Case-Control	<3 years	1,610	1.048 (0.885, 1.255)		4.8	7	0.39
Short-Term Associations With Cardiovascular Hospitalizations										
Atkinson et al. 2010	London, United Kingdom	2000–2005	Time-Series		293,913	1.002 (0.990, 1.014)	1.004 (0.999, 1.008)	7.0	15	0.22
Halonen et al. 2009	Helsinki metropolitan area, Finland	1998–2004	Time-Series		61,571	1.010 (0.998, 1.021)	0.997 (0.985, 1.009)	7.5	9.5	0.25
Host et al. 2008	6 French cities, France	2000–2003*	Time-Series		251,397	1.005 (0.988, 1.023)	1.009 (1.001, 1.018)	7.0–11.0†	13.8–18.8†	0.28–0.73
Peng et al. 2008	108 Counties, United States	1999–2005	Time-Series	>65 years	3.7 M	1.004 (1.001, 1.007)	1.007 (1.005, 1.010)	13.5	9.8	0.12
Qiu et al. 2013	Hong Kong, Special Administrative Region of China	2000–2005	Time-Series		338,123	1.007 (1.000, 1.013)	1.006 (1.003, 1.009)	14.5	34.8	0.68
Stafoggia et al. 2013	6 metropolitan areas, European Mediterranean	2001–2010	Time-Series	>15 years	727,579	1.007 (1.002, 1.013)	1.005 (1.001, 1.009)	9.3–17.5†	17.2–34.4†	0– > 0.5

* Years differed by city, ** PM_1_ reported instead of PM_2.5_, † Mean, †† Dust day, dust-free day

### Associations Between Short-Term PM_10-2.5_ Exposures, Mortality, and Hospital Admissions

**Fig. 1:**
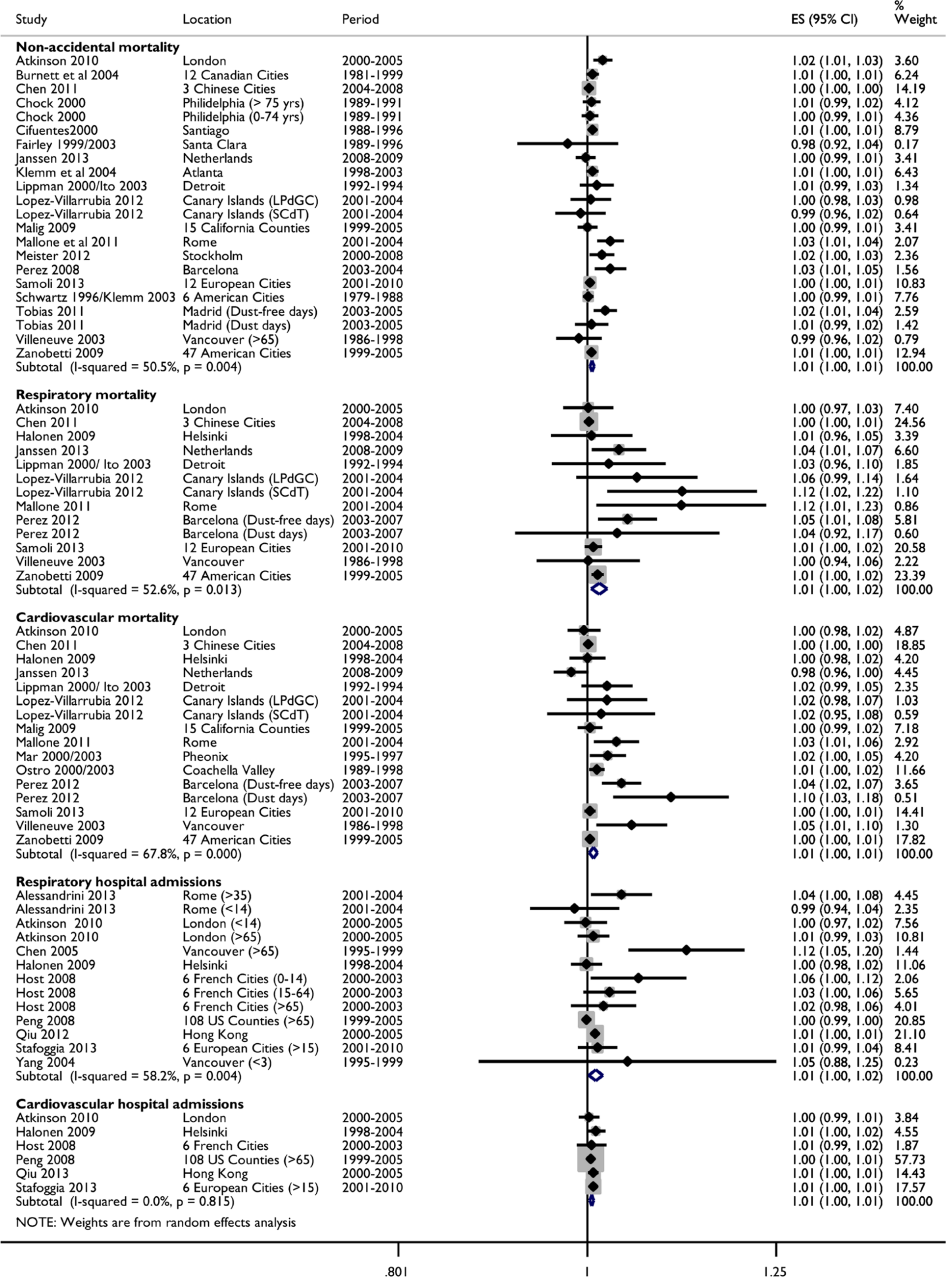
Forest plot of incidence rate ratios for mortality and hospital admissions per 10 μg/m^3^ of short-term exposure to PM_10-2.5_. Note: Overall estimates are from random-effects models without adjustment for possible publication bias

The vast majority of short-term studies linked higher mortality and morbidity with higher PM_10-2.5_ concentrations (Fig. 1). Mortality and hospital admissions due to respiratory causes had the largest associations with random-effects summary estimates of 1.4 % (95 % CI: 0.5–2.4 %) and 1.0 % (95 % CI: 0.1–1.8 %) higher rates per 10 μg/m^3^, respectively (Table 2). These estimates were approximately two to three times higher than the observed associations for total mortality, cardiovascular mortality, and cardiovascular hospital admissions, although the confidence intervals were also much wider. Sensitivity analyses of cause-specific hospital visits (including estimates from emergency department studies) provided consistent evidence of increased rates with increasing levels of PM_10-2.5_ for outcomes including asthma, chronic obstructive pulmonary disease, and ischemic heart disease (results not presented). In general, the inclusion of emergency department visits resulted in a slight weakening of the respiratory but not cardiovascular summary estimates, though the results were qualitatively the same. Exclusion of childhood respiratory admissions also did not substantially alter our findings (results not presented).

**Table 2:** Summary rate ratios (RR) for mortality and hospital admissions per 10 μg/m^3^ of PM_10–2.5_ and PM_2.5_ concentrations

			Short-Term Exposures			Long-Term Exposures
	Total Mortality	Respiratory Mortality	Cardiovascular Mortality	Respiratory Hospitalizations	Cardiovascular Hospitalizations	Total Mortality
Coarse Particulate Matter						
Number of studies	19	11	14	9	6	6
Number of estimates^a^	22	13	16	13	6	6
Pooled RR (95 % CI)^b^	1.006 (1.003–1.008)	1.014 (1.005–1.024)	1.007 (1.002–1.012)	1.010 (1.001–1.018)	1.005 (1.003–1.008)	1.021(0.984–1.058)
Heterogeneity						
I^2^	51 %	53 %	68 %	58 %	0 %	38 %
p-value	0.004	0.013	<0.001	0.004	0.82	0.15
Publication bias						
Adjusted RR (95 % CI)^c^	1.004 (1.001–1.007)	1.007 (0.996–1.018)	1.002 (0.997–1.008)	1.006 (0.996–1.016)	1.005 (1.003–1.007)	0.994 (0.956–1.035)
Egger regression test, p-value	0.05	0.01	0.01	0.07	0.45	0.66
Fine Particulate Matter						
Number of studies	18	11	14	9	7	6
Number of estimates^a^	21	13	15	11	7	6
Pooled RR (95 % CI)^b^	1.007 (1.004–1.009)	1.012 (1.005–1.020)	1.006 (1.004–1.008)	1.009 (1.005–1.013)	1.006 (1.004–1.007)	1.092 (1.009–1.182)
Heterogeneity						
I^2^	75 %	62 %	17 %	27 %	0 %	76 %
p-value	<0.001	0.002	0.26	0.19	0.51	0.001
Publication bias						
Adjusted RR (95 % CI)^c^	1.005 (1.002–1.008)	1.006 (0.998–1.013)	1.006 (1.004–1.008)	1.009 (1.005–1.013)	1.006 (1.004–1.007)	1.061 (0.984–1.143)
Egger regression test, p-value	0.08	0.06	0.20	0.39	0.28	0.32

Notes: ^a^ The number of estimates can differ from the number of studies due to reports stratified by age group and/or Saharan dust days

^b^ Overall estimates are from random-effects models

^c^ Models are adjusted for possible publication bias using a trim and fill approach

Single pollutant associations for PM_10-2.5_ were generally similar to those reported for PM_2.5_ in studies with paired single pollutant estimates (Table 2). Estimates for PM_10-2.5_, however, showed more evidence of possible publication bias as shown by statistically significant findings of asymmetry using Egger’s regression test. Adjustment for asymmetry using a “trim and fill” approach resulted in a weakening, though not elimination, of most associations with PM_10-2.5_. Associations for PM_2.5_ were generally more robust to adjustment for possible publication bias.

**Fig. 2:**
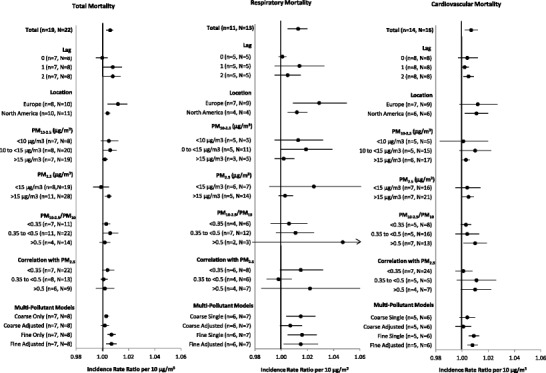
Summary incidence rate ratios for short-term exposures to PM_10-2.5_ with mortality by study characteristics. Note: Estimates stratified by concentrations include city-specific data from Malig and Ostro [35] and Chock et al. [45] provided via personal correspondence. Estimates were also provided by Zanobetti and Schwartz [33••] but ultimately not included because the use of shrunken Bayes estimates could have undue influence on our results

**Fig. 3:**
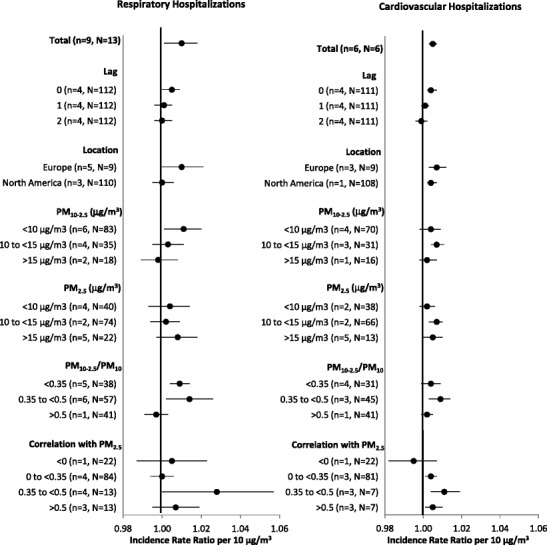
Summary incidence rate ratios for short-term exposures to PM_10-2.5_ with hospital admissions by study characteristics. Note: Estimates stratified by PM concentrations and correlations include city-specific estimates provided by Peng et al. [62••] and Host et al. [61] in personal communications

All outcomes except cardiovascular disease hospital admissions showed moderate (I^2^ = 51–68 %) and statistically significant heterogeneity in the point estimates for PM_10-2.5_ (Table 2). As shown in Figs. 2 and 3, location appeared to be an important explanatory factor for this heterogeneity with stratified analyses indicating that European cities consistently had larger PM_10-2.5_ associations than North America for all outcomes except for cardiovascular mortality. Although there was no clear evidence of heterogeneity by PM_2.5_ concentrations, there was some evidence of lower rate ratios with higher PM_10-2.5_ concentrations for both mortality and hospital admissions. Lower rate ratios were also found when PM_10-2.5_ was more than half of the reported PM_10_ concentrations for hospital admissions but not mortality (meta-regression p-value: 0.06). There was also a suggestion of weaker associations with total mortality among studies using TEOMs and stronger associations among studies using dichotomous samplers but the sample size was small and the differences were not large (results not shown). There were insufficient numbers to examine these relationships with outcomes other than cardiovascular and respiratory mortality and admissions.

**Fig. 4:**
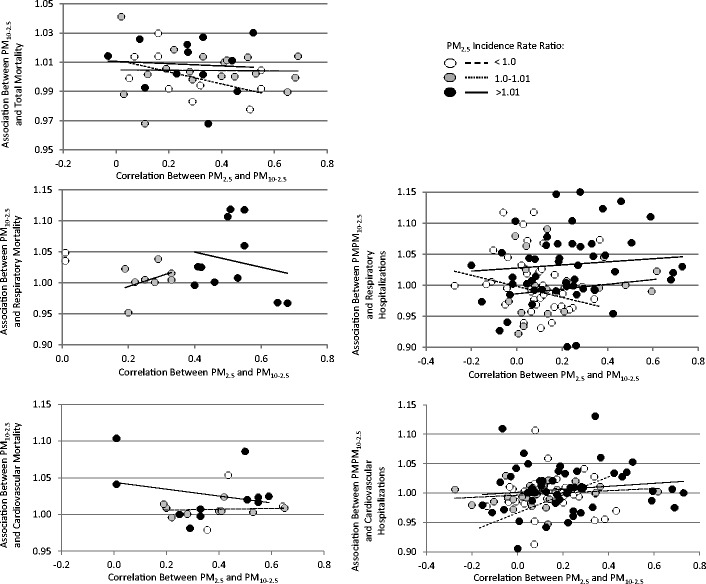
Incidence rate ratios (RR) for PM_10-2.5_ as a function of the correlation between short-term PM_10-2.5_ and PM_2.5_ concentrations stratified by PM_2.5_ associations. Note: Data include city-specific estimates provided by Peng et al. [62••] and Host et al. [61] from personal communications

As shown in Fig. 2, associations between short-term PM_10-2.5_ concentrations and mortality were sensitive to control for PM_2.5_ in two-pollutant models, with a weakening of associations that resulted in a loss of statistical significance in all scenarios. This was especially true for cardiovascular mortality, for which the PM_10-2.5_ association was fully eliminated by control for PM_2.5_ (results not shown). Although there were too few hospital admission studies with multi-pollutant estimates for a formal meta-analysis, these results appeared to be generally less sensitive to control for PM_2.5_. In spite of the observed sensitivity in PM_10-2.5_ associations to control for PM_2.5_, we did not observe a consistent pattern of increasing associations with PM_10-2.5_ with increasing correlations between PM_2.5_ and PM_10-2.5_ concentrations when PM_2.5_ was associated with adverse health (Fig. 4). Nor did we find consistent evidence of smaller associations with PM_10-2.5_ with increasing correlations between PM_10-2.5_ and PM_2.5_ concentrations when PM_2.5_ concentrations were associated with improved health. Associations with PM_2.5_ were less sensitive to control for PM_10-2.5_ concentrations (Fig. 2)

### Papers Identified with Long-Term Exposures to PM_10-2.5_

Estimates of associations between long-term PM_10-2.5_ concentrations and all-cause mortality were available from five American cohort studies [78••, 79, 80••, 81••, 82] and one multicenter study in Europe that combined data from 19 study populations (Table 3) [83]. Additional studies on infant mortality[84] and fatal coronary heart disease [85] were identified but ultimately not included because the number of studies was insufficient to support a meta-analysis. As summarized in Tables 3, these cohort studies collectively followed approximately 780,000 participants over a range of PM_10-2.5_ (4.0 to 27.3 μg/m^3^) and PM_2.5_ concentrations (6.6 to 31.9 μg/m^3^).

**Table 3:** Descriptive information for long-term exposure studies included in the meta-analysis

Study	Location	Time Period	Study Design	Restrictions	Reported or Estimated # of Events (Short-Term) or # of Participants (Long-Term)	Estimated Incidence Rate Ratios (95 % CI) per 10 μg/m^3^ of PM_10–2.5_	Estimated Incidence Rate Ratios (95 % CI) per 10 μg/m^3^ of PM_2.5_	Median or Mean† PM_10–2.5_	Median or Mean† PM_2.5_	Correlation of PM_10–2.5_ and PM_2.5_
Long-Term Associations With Non-Accidental Mortality										
Beelen et al. 2013	19 Cohorts from 12 European Countries	1985–2007	Cohort Studies		327,780	1.08 (0.96, 1.21)	1.14 (1.04, 1.28)	4.0–20.7†	6.6–31.0†	0.11–0.90
Lipfert et al. 2006	32 Veterans Hospitals, United States	1989–1996	Cohort Study	All-Cause	24,642	1.06 (1.01, 1.11)	1.15 (1.05, 1.26)	16†	14.3†	
McDonnell et al. 2000	California, United States	1977–1992	Cohort Study		1,266	1.05 (0.92, 1.20)	1.22 (0.95, 1.58)	27.3†	31.9†	0.5
Pope et al. 2002	50 States, United States	1982–1998	Cohort Study		359,000	1.01 (0.97, 1.05)	1.06 (1.02, 1.11)	19.2†˜	17.7†	
Puett et al. 2009	13 Northeast and Midwest States, United States	1992–2002	Cohort Study		66,250	1.03 (0.89, 1.18)	1.26 (1.02, 1.54)	7.7†	13.9†	
Puett et al. 2011	13 Northeast and Midwest States, United States	1989–2003	Cohort Study		17,545	0.96 (0.91, 1.02)	0.94 (0.87, 1.00)	10.1†	17.8†	

˜ PM_15_ reported instead of PM_10_

### Associations Between Long-Term PM_10-2.5_ Exposures and Mortality

**Fig. 5:**
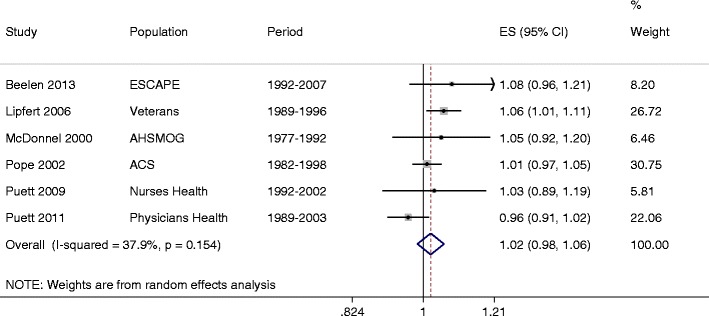
Summary of rate ratios between long-term exposure to PM_10-2.5_ and death per 10 μg/m^3^

Pooled random-effects analyses resulted in a summary estimate of a 2.1 % (95 % CI: -1.6 % to 5.8 %) increased mortality rates per 10 μg/m^3^ higher long-term PM_10-2.5_ concentration (Table 2, Fig. 5). There was limited evidence of heterogeneity among these point estimates (I^2^ = 38 %, p = 0.15) and no finding of publication bias among these five studies. A meta-analysis of multi-pollutant estimates from five studies [79, 80••, 81••, 82, 83] indicated no associations with PM_10-2.5_ after adjustment for PM_2.5_ (-1.2 %, 95 % CI: -5.1 to 2.8 % per 10 μg/m^3^). In contrast, PM_2.5_ associations were weakened after adjustment for PM_10-2.5_ (3.7 %, 95 % CI: 0 to 7.6 % per 10 μg/m^3^) but remained positive and statistically significant. Because there were only six studies identified, we did not investigate stratified analyses by study characteristics.

## Discussion

Although the health implications of PM_10-2.5_ remain far less characterized than those for PM_2.5_, there is a growing epidemiological literature for PM_10-2.5_. In this meta-analysis we identified 23 and 10 studies of short-term associations with mortality and hospitalizations, respectively, as well as 6 papers for long-term associations with mortality. Overall, we found suggestive evidence that higher short-term PM_10-2.5_ concentrations are associated with greater rates of mortality and hospitalizations, with the strongest relationships for respiratory endpoints. There was high heterogeneity in these estimates, however, with stronger associations suggested for European locations as compared to North America and weaker associations for locations with the highest PM_10-2.5_ levels. Adjustments for PM_2.5_ and asymmetry due to possible publication bias resulted in positive associations for PM_10-2.5_ that were weaker and less precise. Higher long-term exposures to PM_10-2.5_ were also associated with larger mortality in single pollutant models but these associations were eliminated by control for PM_2.5_. PM_2.5_ associations in these studies were less sensitive to control for PM_10-2.5_ and had less evidence of asymmetry.

PM_10-2.5_ may plausibly impact health given their deposition in the lungs, high biological content, and, in urban areas, high content of heavy metals.[86] Toxicological studies have provided evidence of the inflammatory effects of PM_10-2.5_, including some evidence that PM_10-2.5_ may be more inflammatory than PM_2.5._[87–93] Controlled human exposure studies have similarly provided some evidence of acute alterations in markers of inflammation, coagulation, and autonomic tone although there was not consistent evidence of stronger associations with PM_2.5_.[94–99] Epidemiological data for subclinical endpoints with PM_10-2.5_ are still relatively sparse but there has been some evidence of biological activity including alterations in cytokines and coagulation factors, pulmonary function, respiratory symptoms, and cardiac function in some [96, 100–106] but not all studies.[104, 107–110] It should be noted, however, that even results from positive studies were often only suggestive and failed to meet statistical significance.

One possible explanation for the inconclusive nature of the literature pertains to the challenges of accurate exposure assessment for PM_10-2.5_. PM_10-2.5_ concentrations are often highly spatially and temporally variable as a consequence of higher deposition velocities as well as the intermittent nature of many PM_10-2.5_ sources.[2] For temporal trends, this has resulted in correlation coefficients between different sites that are generally lower than those reported for PM_2.5_ or PM_10._[111] Concentrations have also been shown to vary across space based on proximity to different sources [112, 113], making long-term exposure assignment especially difficult given the limited numbers of monitoring stations with data to estimate PM_10-2.5_. In addition, most measurements of PM_10-2.5_ are indirect, estimated through subtraction of PM_2.5_ from PM_10_ concentrations measured at the same location. While past research has deemed this a reliable approach to estimating PM_10-2.5_ in urban areas [114], there are inherently errors due to the uncertainty of both filters. Even dichotomous samples for PM_10-2.5_, which are generally thought to have less error due to the use of a virtual impactor, may also have additional uncertainty due the small deposition of PM_2.5_ in the PM_10-2.5_ channel [115]. Similarly, continuous monitors such as the TEOM have been shown to be subject to measurement error if the losses of semi-volatile material are not properly accounted for [13]. Finally, infiltration rates for PM_10-2.5_ are quite low in comparison to PM_2.5_ and the presence of indoor sources are high, suggesting that ambient exposure may not accurately estimate personal exposure [116].

Although we only had limited data to investigate the impacts of measurement error on associations with health, we found some evidence of its importance with stronger associations among short-term concentrations measured using dichotomous samplers as compared to difference metrics, and weaker associations in studies using TEOMs as compared to other techniques. The three investigations using spatial prediction models to assess small-scale variability of long-term PM_10-2.5_ concentrations, however, did not consistently have stronger associations with mortality than other investigations relying only on central monitoring stations. Given these challenges for the measurement of PM_10-2.5_, we encourage researchers to be mindful of the methods used to assess exposure and report on the potential implications for their analyses. Epidemiological research is underway as part of the Colorado Course Rural Urban Sources and Health Study [117] for short-term exposures and the Multi-Ethnic Study of Atherosclerosis and Coarse Particulate Matter (MESA Coarse) [112] for long-term exposures that incorporates more accurate estimates of exposure, and thus should be subject to less measurement error.

Larger measurement error relative to PM_2.5_ may be a plausible explanation for the weakened associations for PM_10-2.5_ in two-pollutant models. First, the presence of greater classical measurement error is likely to result in a reduction of the point estimate towards the null. In addition, it has been hypothesized that a transfer of association from a variable with more measurement error to another with less error may occur in situations where there are substantial differences in the measurement error [118]. Another explanation is that confounding is present, although PM_2.5_ and PM_10-2.5_ concentrations only exhibited modest correlations in the incorporated studies (range: 0.0–0.7, median ~ 0.3). Furthermore, there was no consistent evidence of increasing associations for PM_10-2.5_ with increasing correlations between PM_2.5_ and PM_10-2.5_ concentrations when PM_2.5_ was associated with a worsening of health. Nor did we find consistent evidence of decreasing PM_10-2.5_ associations with increasing correlations between PM_10-2.5_ and PM_2.5_ concentrations when PM_2.5_ was found to be protective of health. Thus, while it may be compelling to assume that any observed associations with PM_10-2.5_ are due to PM_2.5_, our results do not support this as the sole explanation. Nevertheless, we encourage future investigations to continue exploring multi-pollutant models and reporting correlations between pollutants to better understand these complex relationships.

While it does not appear as though associations with PM_10-2.5_ are simply due to confounding by PM_2.5_, it remains possible that both PM_2.5_ and PM_10-2.5_ are acting as surrogates of a broader mixture of pollution. Thus, it may be that another unmeasured component or several components are the true causal factors. For example, in rural areas, gram-negative bacteria (as represented by bacterial-derived lipopolysaccharide or endotoxin) PM_10-2.5_ may be of special interest, especially for inflammatory mechanisms [87, 88, 97]. In urban areas, metals associated with roadway dust may be similarly important [89, 91, 119, 120]. The general lack of investigation of endotoxin levels, components of PM_10-2.5_, and multi-pollutant mixtures remains a weakness of the existing literature and an area for future development.

Along similar lines, it has been hypothesized that the toxicity of PM_10-2.5_ may be greater for particles originating in urban environments as compared to rural environments. Some evidence of such a relationship has been reported in 108 US counties [62••] and at least one toxicology study [88]. In this meta-analysis, we found evidence that PM_10-2.5_ associations with health were often weaker in regions with higher levels of PM_10-2.5._ This may suggest a non-linear dose response, as was reported in China [63••], or a difference in toxicity for more rural or arid regions. Weaker associations between PM_10-2.5_ and hospital admissions in regions with higher PM_10-2.5_/PM_10_ ratios may also support different toxicity by region, but the same pattern was not robust for morality. Interestingly, several investigators have attempted to distinguish toxicity of particulate matter from dust storms, but uncertainty remains around this question. Among those studies included in this meta-analysis, larger associations between short-term PM_10-2.5_ and health were reported on Saharan dust days in Rome [41, 59], whereas results with mortality in Madrid and Barcelona stratified by dust days were more mixed [31, 46]. While additional research may be needed from rural locations to inform this question, challenges will always remain unless speciated data is used, since anthropogenic and biological particles likely adhere to dust particles as they are transported through other airsheds.

Overall, this work adds to the literature by presenting the first meta-analysis results for PM_10-2.5_. With numerous new investigations in the literature, we also conducted stratified analyses to explore differences in associations with hospital admissions and mortality by various characteristics of the locations studied. As substantial heterogeneity was present among the associations presented, this represents an important area that requires further exploration in future investigations. In fact, it should be noted that the summary estimates reported in this analysis should be viewed with caution due to the presence of heterogeneity. Likewise, the observed heterogeneity suggests that the trim and fill method used to account for potential publication bias may be an overly conservative approach. While it may be challenging to fully characterize different personal characteristics that confer susceptibility, or components of the air pollution mixture that may lead to greater risk of morbidity and mortality in time-series studies, other designs not included in this investigation such as panel studies and controlled clinical studies have important contributions to make.

## Conclusions

Suggestive evidence was observed for increased hospital admissions and mortality with higher levels of short-, but not long-term, PM_10-2.5_ concentrations. Relationships were generally stronger for respiratory endpoints, though associations with cardiovascular endpoints could not be excluded. Similarly, in spite of some sensitivity of the associations to control for PM_2.5,_ our analysis suggests that associations with short-term exposures to PM_10-2.5_ cannot be fully explained by confounding by PM_2.5_. Additional research is still required to better understand sources of heterogeneity in associations, including co-exposure with other pollutants, sources, spatial variability, and composition of PM_10-2.5_, as well as individual susceptibilities.
